# Hippocampal Endoplasmic Reticulum Stress Hastens Motor and Cognitive Decline in Adult Male Rats Sustainedly Exposed to High-Sucrose Diet

**DOI:** 10.3390/antiox11071395

**Published:** 2022-07-19

**Authors:** Bruno Araújo Serra Pinto, Thamys Marinho Melo, Karla Frida Torres Flister, Lucas Martins França, Vanessa Ribeiro Moreira, Daniela Kajihara, Nelmar Oliveira Mendes, Silma Regina Pereira, Francisco Rafael Martins Laurindo, Antonio Marcus Andrade Paes

**Affiliations:** 1 Laboratory of Experimental Physiology, Department of Physiological Sciences, Federal University of Maranhão, Av. dos Portugueses 1966, Bacanga, São Luís 65080-805, MA, Brazil; bruno.pinto@ufma.br (B.A.S.P.); thamys.marinho@discente.ufma.br (T.M.M.); karla.flister@ufma.br (K.F.T.F.); lucas.mf@ufma.br (L.M.F.); nelmar.mendes@discente.ufma.br (N.O.M.); 2 Laboratory of Genetics and Molecular Biology, Department of Biology, Federal University of Maranhão, Av. dos Portugueses 1966, Bacanga, São Luís 65080-805, MA, Brazil; more_nessa@yahoo.com.br (V.R.M.); silma.pereira@ufma.br (S.R.P.); 3 Laboratory of Vascular Biology, Heart Institute of the School of Medicine, University of São Paulo, Av. Dr. Enéas Carvalho de Aguiiar, 44, Cerqueira César, São Paulo 05403-900, SP, Brazil; d.kajihara@hc.fm.usp.br (D.K.); francisco.laurindo@incor.usp.br (F.R.M.L.)

**Keywords:** high-sucrose diet, metabolic syndrome, hippocampus, ER stress, cognitive and motor impairments

## Abstract

Metabolic dysfunctions, such as hyperglycemia and insulin resistance, have been associated to cognitive impairment and dementia regardless of advanced age, although the underlying mechanisms are still elusive. Thus, this study investigates the deleterious effects of metabolic syndrome (MetS) induced by long-term exposure to a high-sucrose diet on motor and cognitive functions of male adult rats and its relationship with hippocampal endoplasmic reticulum (ER) stress. Weaned Wistar male rats were fed a high-sucrose diet until adulthood (HSD; 6 months old) and compared to both age-matched (CTR; 6 months old) and middle-aged chow-fed rats (OLD; 20 months old). MetS development, serum redox profile, behavioral, motor, and cognitive functions, and hippocampal gene/protein expressions for ER stress pro-adaptive and pro-apoptotic pathways, as well as senescence markers were assessed. Prolonged exposure to HSD induced MetS hallmarked by body weight gain associated to central obesity, hypertriglyceridemia, insulin resistance, and oxidative stress. Furthermore, HSD rats showed motor and cognitive decline similar to that in OLD animals. Noteworthy, HSD rats presented marked hippocampal ER stress characterized by failure of pro-adaptive signaling and increased expression of Chop, p21, and Parp-1 cleavage, markers of cell death and aging. This panorama resembles that found in OLD rats. In toto, our data showed that early and sustained exposure to a high-sucrose diet induced MetS, which subsequently led to hippocampus homeostasis disruption and premature impairment of motor and cognitive functions in adult rats.

## 1. Introduction

Metabolic syndrome (MetS) is a worldwide alarming public-health concern related to excess energy intake associated to a sedentary lifestyle, which leads to metabolic dysfunctions highly associated with increased risk of cardiovascular diseases, type 2 diabetes mellitus (T2DM), and all-cause mortality [1,2]. Clinical criteria for MetS demands the presence of at least three out of the following: insulin resistance (IR), central adiposity, impaired glycemic profile, atherogenic dyslipidemia, and hypertension [3]. Nevertheless, recent studies have increasingly shown an association between MetS-associated dysfunctions, especially IR, with impaired cognitive function and dementias, regardless of advanced age [4,5], but seemingly related to early MetS onset [6].

The hippocampus is classically responsible for cognitive processing, but its role has been expanded by studies showing that neurons projecting from the hippocampal ventral region toward the amygdala and ventral striatum are involved in motor and behavioral modulation [7,8]. The hippocampus also acts as a neuroendocrine interconnector between metabolic signals and cognitive functions, particularly in obesity models based on dietary fructose [9,10]. Currently, it is well-established that metabolic dysfunctions, such as hyperglycemia and IR, are directly involved in cellular senescence [11] through the disruption of key homeostatic processes, such as apoptosis, autophagy, and neurogenesis, which are potentially shared by aging- and obesity-triggered mechanisms [6,12]. Although the underlying signaling pathways are still elusive, endoplasmic reticulum (ER) stress has emerged as a potential linking mechanism. This assumption is supported by recent demonstrations that cognitive function is impaired by ER stressors like deltamethrin [13] and sevoflurane [14] but improved by the administration of 4-phenyl butyrate, a chemical chaperone, to aged rats [15].

ER stress can be defined as an unbalance between misfolded proteins’ overload in the ER lumen and its limited capacity to handle such aberrant proteins [16]. To control ER stress, a complex network of adaptive signaling, known as the unfolded protein response (UPR), is activated to re-establish ER homeostasis and ensure cell survival [17]. However, under persistent unsolved ER stress, UPR migrates from an adaptive to a pro-apoptotic pattern that ultimately leads to cell death [18]. More recently, ER stress has consolidated its role as a key feature of metabolic disorders [19]. Aging, in a similar way, leads to impaired ER chaperoning that causes improper protein folding [20,21]. Likewise, several neurodegenerative age-related diseases have been associated with aberrant protein accumulation, particularly Alzheimer’s and Parkinson’s diseases [22].

Therefore, we have hypothesized that hippocampal ER stress-activated pathways underlie the interconnection between long-term metabolic dysfunctions and neurofunctional decline. In a previous report, we have shown that MetS induced by post-weaning exposure to a high-sucrose diet triggers hippocampal ER stress pathways leading to motor and behavioral but not cognitive impairments in male rats as young as 3 months old [23]. Thus, in the present study, we showed that long-term exposure to the same diet until 6 months old deepened ER stress-driven hippocampal damage and hastened cognitive decline in a way very similar to that found in 20-month-old chow-fed rats. These data emphasize the dysfunctional outcomes of early MetS onset on neuromotor and cognitive health of young-to-adult subjects.

## 2. Materials and Methods

### 2.1. Animals and Experimental Design

Weaned male Wistar rats provided by the animal facility house of the Federal University of Maranhão (postnatal day (pnd) 21, 51.07 ± 0.57 g) were randomized into two groups: control rats (CTR; n = 7) fed a standard chow (Nuvital^®^, Nuvilab, Curitiba, Brazil) composed of 55.4% total carbohydrate (10% sucrose), 21% proteins, 5.2% total lipids, totaling 352.17 kcal·100 g^−1^, or high-sucrose diet rats (HSD, n = 7) fed a high-sucrose chow composed of 65% total carbohydrates (25% sucrose), 12.3% proteins, 4.3% total lipids, totaling 348.16 kcal·100 g^−1^, as previously described in [23]. Both groups were followed for 24 weeks until 6 months old. An additional group of 20-month-old rats that had always been fed a standard chow (OLD, n = 7) was introduced one week before the end of the experimental period and used as a neuronal aging control for hippocampus-driven molecular and functional assessments. The animals were maintained in a controlled room (21 ± 2 °C; 60% humidity and 12 h light/dark cycle) with water and chow *ad libitum*. 

Throughout the dietary interventional period, body weight and energy intake were assessed twice a week. The Lee index (body weight (g)^1/3^·naso-anal length (cm)^−1^·1000) was calculated every month for assessment of obesity development [24]. Behavioral and cognitive tests were performed during the last week before euthanasia. In parallel, the animals were also subjected to intraperitoneal glucose and insulin tolerance tests. At pnd 180, overnight-fasted animals were anesthetized (40:10 mg/kg ketamine–xylazine solution) for blood and tissue collection upon laparotomy, as well as craniotomized for hippocampus dissection. Retroperitoneal, periepididymal, and mesenteric white adipose tissue fat pads, liver, and posterior skeletal muscles (gastrocnemius and soleus) were weighed for morphometric assessment and expressed as tissue mass (g) per 100 g of body weight. Liver samples were also used for fat liver content measurement. Serum samples were used for the assessment of biochemical, hormonal, and redox profiles, whereas hippocampi homogenates were used for gene/protein expression protocols. All procedures were performed in accordance with the rules of the Brazilian Council for the Control of Animal Experimentation (CONCEA) and approved by the Ethical Committee on Animal Use and Welfare of the Federal University of Maranhão, under ruling number 23115.009595/2014-81.

### 2.2. Assessment of Liver Fat Content

Liver fat accumulation was assessed from 500 mg liver samples, as previously described [25]. Briefly, the samples were homogenized with a chloroform/methanol solution (2:1), and the resulting supernatant was paper-filtered and diluted in 0.9% NaCl (5:1). The organic phase was collected, air-flow oven-dried at 40 °C, and weighed to determine the total fat content (mg) per tissue mass (g). The total fat was resuspended in Triton X/methanol (2:1) for colorimetric measurement of triglycerides and total cholesterol. The results were expressed as triglycerides (mg) per tissue mass (g) and total cholesterol (mg) per tissue mass (g).

### 2.3. Assessment of Glucose-Insulin Axis Function

For an intraperitoneal glucose tolerance test (*ip*GTT), the animals were submitted to 8 h of fasting prior to administration of 2 g/kg of glucose. Tail vein blood drops were collected immediately before (time 0) and 15, 30, 60, and 120 min after a glucose bolus for glucose measurement through a glucometer (Accucheck Active^®^, Roche Diagnostic, Mannheim, Germany). The data were expressed as area under glycemic curve (AUC). A similar procedure was carried out for an intraperitoneal insulin tolerance test (*ip*ITT), except the animals were fed and received 1 UI/kg of insulin (Humulin 70/30^®^, Lilly, Indianapolis, IN, USA). The glucose disappearance rate (kITT) was derived from the ITT curve and calculated as 0.693/t_1/2_ [26]. Peripheral insulin sensitivity was inferred by calculation of the homeostasis model assessment (HOMA) Index (HOMA-IR = fasting glucose (mM)·fasting insulinemia (µU/mL)·22.5^−1^) and the HOMA Index of β-cell function (HOMA-B = (fasting insulin (µU/mL)·20)·(fasting glucose (mM) − 3.5)^−1^ [27]. Hepatic IR was inferred from TyG index calculation [ln·(fasting glucose (mg/dL)·fasting triglyceride (mg/dL))·2^−1^] [28]. 

### 2.4. Assessment of Serum Biochemical and Redox Profiles

Serum samples were used for colorimetric measurement of triglycerides, total cholesterol (Labtest, Lagoa Santa, MG, Brazil), and free fatty acids’ (Sigma-Aldrich, Saint Louis, MO, USA) levels according to the manufacturer’s instructions. Insulin levels were assessed by an immunoassay (Sigma-Aldrich, Saint Louis, MO, USA). The redox profile was assessed by measuring antioxidant enzymes’ activities, namely those of superoxide dismutase (SOD), catalase (CAT) and gluthathione peroxidase (GPx), according to the kits manufacturers’ instructions (Cayman Chemical, Ann arbor, MI, USA), as were the serum malondialdehydes levels (MDA) [29].

### 2.5. Assessment of Behavioral, Cognitive, and Motor Functions

In order to assess neuromotor activity, rotarod equipment was used. Briefly, animals were accommodated on a rod and trained 3 times a day for 4 consecutive days. At day 5, the animals were fasted for 2 h before experimental performance, when the rod was set at 12 rpm and the latency to fall was recorded in seconds for a maximum of 5 min [30]. To assess exploratory and anxiety-like behavior, the animals were individually placed at the center of an open-field circular arena (140 cm^2^) divided into 9 quadrants for 5 min. The total number of quadrant crossings, frequency of entrances in the central and peripheral quadrants, and stereotyped behaviors were recorded for subsequent analysis [31]. 

To assess spatial learning/memory functions, the animals were subjected to a water maze test [32]. The apparatus consisted of a circular fiberglass pool filled with water and divided into four quadrants with presence of visible cues on the walls for the rats. A hidden escape platform was submerged so that it was 1.5 cm below the water surface in the pre-set quadrant. The animals were subjected to 4 trials per day during 3 consecutive days, when they were individually released into the water from one of the four quadrants, facing a maze wall, and allowed to swim for 120 s to find the hidden platform. Rats that failed to locate the platform were gently guided to the platform and allowed to stay on it for 30 s. Latency to find the hidden platform was recorded for spatial learning assessment and expressed as the mean time from the daily trials. At day 4, the platform was removed, and the animals were released in the center of the pool to swim for 120 s. The time spent in the quadrant where platform was expected to be, as well as the proportional number of entries in the target quadrant, were recorded for memory retention assessment.

### 2.6. Gene Expression by Real-Time PCR (qPCR)

Hippocampi samples (n = 5 per group) were used for RNA extraction using Trizol^®^ (Invitrogen, Berlin, Germany) as per the manufacturer’s instructions. RNA samples (3 μg) were converted into cDNA using Super Script II Reverse Transcriptase^®^ (Invitrogen, Carlsbad, CA, USA). qPCR amplification and Platinum^®^ SYBR^®^ Green qPCR SuperMix-UDG (Invitrogen, Carlsbad, CA, USA) detection were performed using 7500 Realtime PCR Applied Biosystems, Waltham, MA, USA. The reactions were incubated at 50 °C for 2 min and at 95 °C for 2 min followed by 40 cycles of 95 °C for 15 s and 60 °C for 1 min. For the melt curve stage, the reactions were incubated at 95 °C for 15 s, 60 °C for 60 min, and 95 °C for 15 s. The primers were designed using Primer Express^®^ software (Applied Biosystem, USA) and manufactured by Invitrogen (Belo Horizonte, MG, Brazil (Supplementary Table S1). All samples were normalized to the relative levels of GAPDH, and the results were expressed as the fold change (FC) values of 2^−ΔΔCT^, as determined by real-time amplification.

### 2.7. Protein Levels by Immunoblotting

Hippocampi samples (n = 5 per group) were homogenized in a lysis buffer containing protease inhibitors (1 μg/mL aprotinin, 1 μg/mL leupeptin, and 10 mM PMSF). After protein quantification by the Bradford reagent method, aliquots (30 μg) were diluted in a sample buffer, subjected to 12% SDS–PAGE, and transferred to nitrocellulose membranes. Detection of specific proteins was performed by incubating membranes with primary antibodies (anti-Kdel (1:1000, Enzo Life Sciences, Farmingdale, NY, USA), anti-Pdi (1:1000, Thermo Fisher Scientific, Waltham, MA, USA), anti-Chop (1:1000, Thermo Fisher Scientific, Waltham, MA, USA) and anti-Parp-1 (1:2500, Calbiochem, San Diego, CA, USA), followed by peroxidase-conjugated secondary antibodies and revealed by the chemiluminescence method (peroxidase-H_2_O_2_-luminol). The results were expressed as relative density. An equal amount of protein in each lane was confirmed by hybridization with anti-β-Actin (1:1000, Sigma Aldrich, Saint Louis, MO, USA).

### 2.8. Statistical Analysis

The sample size was calculated by using the free software G* Power 3.1 [33] on the basis of the effects of HSD on body weight gain and plasma parameters from a previous study [23]. A minimal sample size of five animals per group was found to provide the appropriate power (1 − β = 0.8) to identify significant differences (α = 0.05) in the variables analyzed. Statistical analysis was conducted using GraphPad Prism 7.0 software (GraphPad Software Inc., San Diego, CA, USA). The data were expressed as the mean ± SEM and submitted for a normality test (Kolmogorov–Smirnov) followed by parametrical analysis through an unpaired t-test (one-tailed) or one-way ANOVA (post-test Newman–Keuls) for a significance level of 5% (*p* < 0.05).

## 3. Results

### 3.1. Long-Term Exposure to a High-Sucrose Diet Induces the Metabolic Syndrome Phenotype in Adult Rats

**Figure 1:**
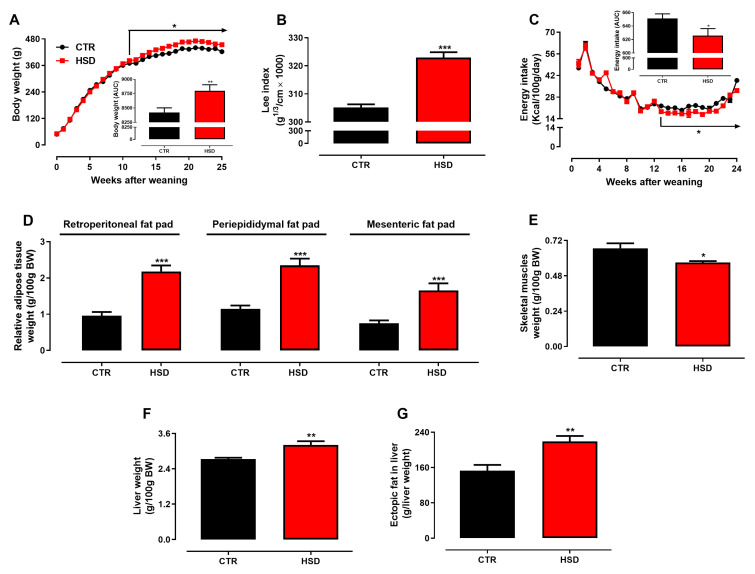
**Morphometric parameters.** (**A**) Body weight (BW, g) and AUC, (**B**) Lee index (g^1/3^ ∙ cm^−1^ × 1000), (**C**) energy intake (Kcal/100g/day) and AUC, (**D**) relative weight (g/100g BW) of retroperitoneal fat, periepididymal fat, mesenteric fat, (**E**) skeletal muscles, (**F**) liver, and (**G**) total fat accumulation in the liver (g/liver weight (g)), assessed in rats fed a standard chow (CTR, n = 7) and a high-sucrose diet (HSD, n = 7) until 6 months from weaning. Points and bars represent the mean ± SEM (unpaired *t*-test one-tailed). * *p* < 0.05, ** *p* < 0.01, *** *p* < 0.001.

Early and long-term feeding of weaned rats with a high-sucrose diet induced MetS-related disorders assessed at adulthood (6 months old) mainly characterized by increased body weight associated to a higher Lee index, regardless of reduced caloric intake in HSD rats, as compared to CTR (Figure 1A–C). HSD rats showed increased visceral and non-visceral fat pads’ accumulation (Figure 1D), whereas their posterior skeletal muscles were atrophied (Figure 1E). Additionally, HSD presented weightier livers (Figure 1F) containing expressively more ectopic fat (Figure 1G).

**Figure 2:**
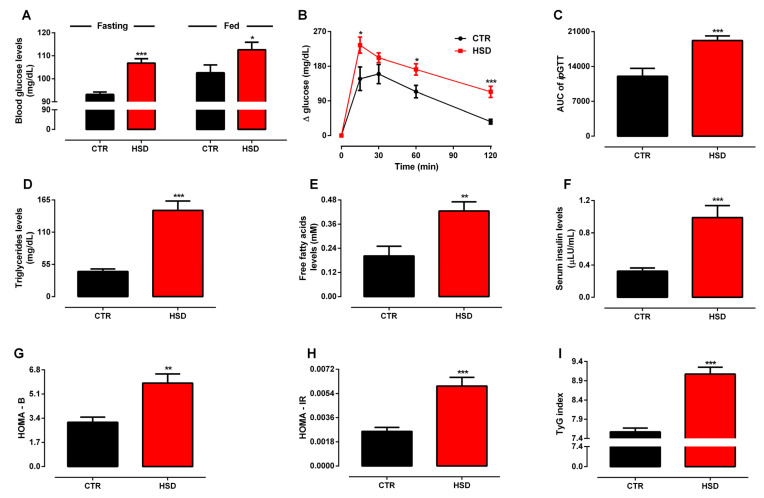
**Glucose–insulin axis function assessment.** (**A**) Fasting and fed blood glucose levels (mg/dL), (**B**) blood glucose levels (mg/dL) during an intraperitoneal glucose tolerance test (ipGTT), (**C**) AUC of ipGTT, (**D**) serum triglyceride levels (mg/dL), (**E**) serum free fatty acids (mM), (**F**) serum insulin levels (μLU/mL), (**G**) HOMA-B, (**H**) HOMA-IR, (**I**) TyG index assessed in rats fed a standard chow (CTR, n = 7) and a high-sucrose diet (HSD, n = 7) until 6 months from weaning. Points and bars represent the mean ± SEM (unpaired *t*-test one-tailed). * *p* < 0.05, ** *p* < 0.01, *** *p* < 0.001.

In accordance with the obese phenotype, HSD rats presented higher blood glucose levels in both fasting and fed states (Figure 2A), which was consistent with their impaired glucose tolerance, as assessed by the *ip*GTT test (Figure 2B,C). The insulin-induced glucose disappearance rate (*k*ITT) did not differ between groups (data not shown), whereas HSD rats were hyperinsulinemic in comparison to CTR rats (Figure 2F). The impairment of insulin homeostasis was further supported by the calculation of HOMA Indexes, which suggested increased secretory capacity of pancreatic β-cells (Figure 2G) and lower peripheral insulin sensitivity (Figure 2H) induced by long-term HSD feeding. Serum triglycerides and free fatty acid levels were augmented by nearly 241.7% and 109.9%, respectively, in the HSD group (Figure 2D,E), while no differences were detected for total cholesterol and its fractions (data not shown). Finally, as an attempt to associate triglyceride production with hepatic insulin sensitivity, calculation of the TyG index showed that the HSD group presented higher values (Figure 2I), which was consistent with hepatic IR. Given the broad age difference between CTR/HSD (6 months old) and OLD (20 months old) groups, which made them incomparable, the morphometabolic profile of the latter was not fully assessed. Notwithstanding, as shown in Supplementary Table S2, OLD rats were normoglycemic in both fasting and fed states, despite increased adipose tissue accumulation.

In whole, this set of data supports that early and long-term exposure to a high-sucrose diet induces MetS, mainly characterized by central obesity, disturbance of the glucose–insulin axis, and peripheral as well as hepatic IR.

### 3.2. Metabolic Syndrome Induced by High-Sucrose Consumption Promotes Serum Redox Unbalance and Oxidative Damage

**Figure 3:**
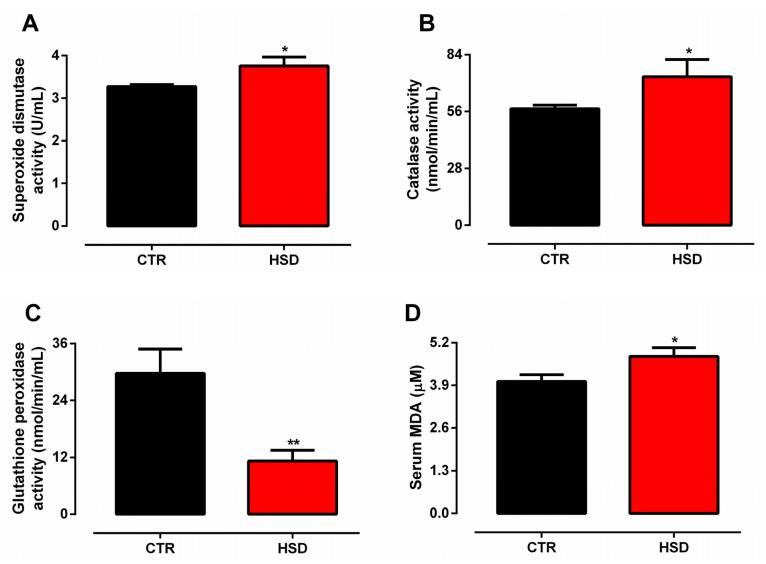
**Serum redox profile.** (**A**) Superoxide dismutase activity (U/mL), (**B**) catalase activity (nmol/min/mL), (**C**) glutathione peroxidase activity (nmol/min/mL), (**D**) serum MDA (μM) assessed in rats fed a standard chow (CTR, n = 7) and a high-sucrose diet (HSD, n = 7) until 6 months from weaning. Bars represent the mean ± SEM (unpaired t-test one-tailed). * *p* < 0.05, ** *p* < 0.01.

Assessment of the serum redox profile showed that the HSD group had SOD and CAT activities increased by approximately 15% and 28%, respectively (Figure 3A,B), whereas GPx activity was reduced by nearly 62% (Figure 3C). Accordingly, HSD rats presented higher lipid peroxidation, as demonstrated by a 20% increase of MDA levels in comparison to those of CTR (Figure 3D).

### 3.3. Metabolic Syndrome Induced by High-Sucrose Consumption Impairs Motor, Behavioral, and Cognitive Functions

**Figure 4:**
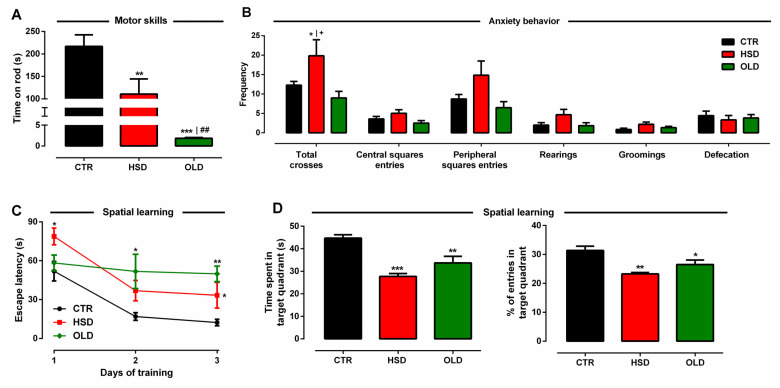
**Motor, behavioral, and cognitive assessments.** (**A**) Motor skills’ assessment by time on rod (s) in a rotarod test, (**B**) behavioral assessment by frequency of total crosses, central and peripheral squares entries, rearings, groomings, and defecation in an open-field test, (**C**) spatial learning assessment by latency time (s) to find the hidden platform in a water maze test, (**D**) memory assessment by time spent (s), as well as the number of entries in the target quadrant in a water maze test assessed in rats fed a standard chow (CTR, n = 7) and a high-sucrose diet (HSD, n = 7) until 6 months from weaning and in middle-aged (20 months old) animals fed a standard chow (OLD, n = 7). Points and bars represent the mean ± SEM (one-way ANOVA Newman–Keuls). * *p* < 0.05, ** *p* < 0.01, *** *p* < 0.001 when compared to CTR, ^+^ *p* < 0.05 when compared to OLD, and ^##^ *p* < 0.01 when compared to HSD.

In order to verify central repercussions of the MetS phenotype induced by early and long-term exposure to high-sucrose consumption, both CTR and HSD rats were subjected to neurofunctional tests with additional comparison with the performance of the OLD group. In the rotarod test, time spent on the rod was decreased by 48.9% and 99.1% in HSD and OLD groups, respectively (Figure 4A), depicting a substantial motor deficit. Meanwhile, the animals’ performance in the open-field test revealed increased anxiogenic behavior in the HSD group, characterized by 62% higher ambulation, an outcome not observed in OLD rats when both groups were compared to CTR (Figure 4B). Nevertheless, other anxiety-related stereotyped behaviors were not observed in any group.

Cognitive capacity, represented by spatial learning and memory retention, was assessed through the water maze test. HSD rats spent more time to find the hidden platform throughout the three-day trial period, as compared to that of CTR (Figure 4C). Noteworthy, OLD rats did not show the same adaptive behavior since the time spent to find the platform did not decay in the second and third days. On the fourth day, memory assessment displayed significant impairment of memory consolidation in both HSD and OLD rats, since these animals spent, respectively, 38% and 25% less time on the correct quadrant in relation to that of CTR (Figure 4D). Accordingly, HSD and OLD animals also did not enter the target quadrant as much as CTR animals did, (Figure 4E), an indication of their increased time swimming away from their target.

### 3.4. Failure of Pro-Adaptive and Activation of Pro-Apoptotic Pathways Anticipate Hippocampal Senescence in Cognitively Deficient HSD Rats

**Figure 5:**
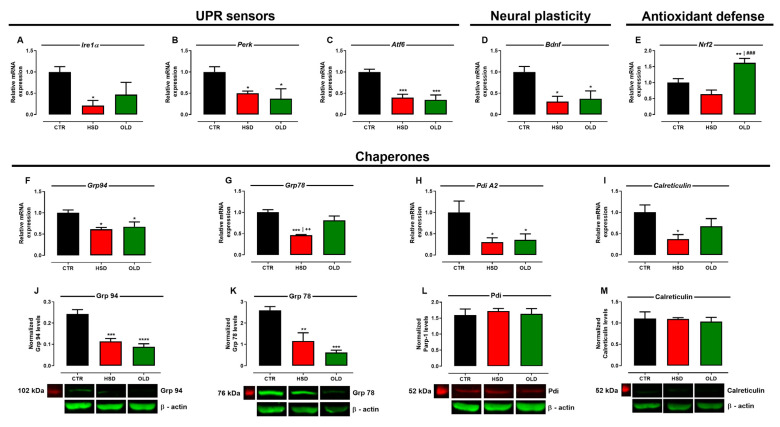
**Expression of UPR pro-adaptive markers in hippocampus.** Relative mRNA expression of UPR sensors Ire1α (**A**), Perk (**B**), and Atf6 (**C**), neural plasticity Bdnf (**D**), antioxidant defense Nrf2 (**E**), and chaperones Grp94 (**F**), Grp78 (**G**), PdiA2 (**H**), and Calreticulin (**I**); and protein levels of chaperones Grp94 (**J**), Grp78 (**K**), Pdi (**L**), and Calreticulin (**M**) assessed in the hippocampi of rats fed a standard chow (CTR, n = 5) and a high-sucrose diet (HSD, n = 5) until 6 months from weaning and middle-aged (20-months-old) animals fed a standard chow (OLD, n = 5). Gene expressions were normalized to the relative levels of GAPDH, the results are expressed as the fold change values of 2^−ΔΔCT^, and protein levels are expressed as relative density normalized by anti-β-Actin. Bars represent the mean ± SEM (one-way ANOVA Newman–Keuls). * *p* < 0.05, ** *p* < 0.01, *** *p* < 0.001, **** *p* < 0.0001 when compared to CTR, ^++^ *p* < 0.01 when compared to OLD, and ^###^ *p* < 0.001 when compared to HSD.

The hippocampus’ role is not restricted to cognitive functions but rather influences other brain areas responsible for metabolic, motor, and behavioral control [34]. Thus, we next investigated whether the disruption of hippocampus’ homeostasis underlies the cognitive impairment observed in HSD as well as OLD rats. Assessment of several markers involved in pro-adaptive UPR signaling showed reduced gene expression of UPR sensors (Ire1α, Perk, and Atf6) in both HSD and OLD groups to levels lesser than half that found in the CTR group (Figure 5A–C). Gene expression of ER chaperones (Grp94, Grp78, PdiA2, and Calreticulin) was similarly reduced in the HSD group, while only Grp94 and PdiA2 were decreased in OLD rats (Figure 5F–I), a finding further supported by decreased protein levels of chaperones Grp94 and Grp78 in the same groups (Figure 5J–M). Bdnf, a factor related to neural plasticity, was significantly less-expressed in HSD and OLD rats (Figure 5D), meanwhile, expression of Nrf2, a nuclear factor related to antioxidant defense, was unchanged in HSD but increased in OLD rats (Figure 5E).

**Figure 6:**
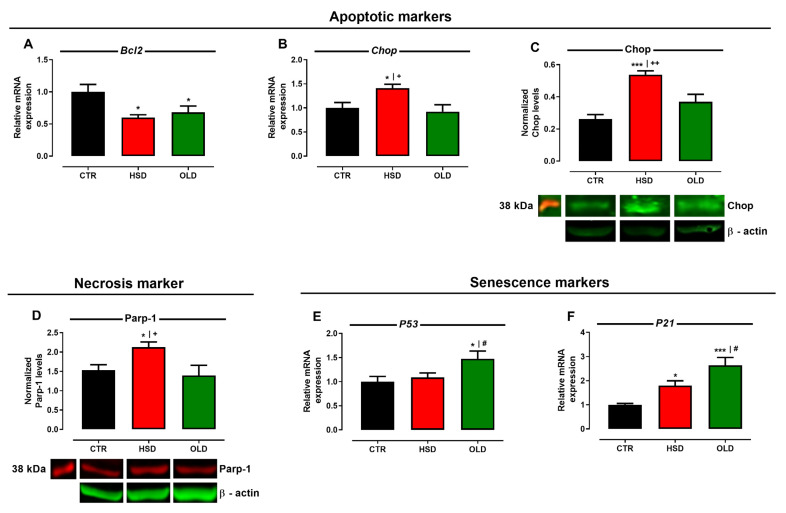
**Expression of cell death and senescence markers in the hippocampus.** Relative mRNA expression of the anti-apoptotic marker Bcl2 (**A**), gene expression (**B**), and protein levels (**C**) of the pro-apoptotic marker Chop; Calpain presence inferred by PARP-1 protein levels (**D**), relative mRNA expression of senescence markers p53 (**E**) and p21 (**F**) assessed in the hippocampi of rats fed a standard chow (CTR, n = 5) and a high-sucrose diet (HSD, n = 5) until 6 months from weaning, and middle-aged (20 months old) animals fed a standard chow (OLD, n = 5). Gene expressions were normalized to the relative levels of GAPDH, the results are expressed as the fold change values of 2^−ΔΔCT^, and protein levels are expressed as relative density normalized by anti-β-Actin. Bars represent the mean ± SEM (one-way ANOVA Newman–Keuls). * *p* < 0.05, *** *p* < 0.001 when compared to CTR, ^+^ *p* < 0.05, ^++^ *p* < 0.01 when compared to OLD, and ^#^ *p* < 0.05 when compared to HSD.

Regarding the triggering of UPR pro-apoptotic pathways, hippocampal gene expression of Bcl2, an important anti-apoptotic factor, was reduced in HSD and OLD groups (Figure 6A); an effect oppositely corroborated by increased gene expression (Figure 6B) and protein levels (Figure 6C) of Chop, an apoptotic factor, in HSD, but not in OLD rats. In addition, Parp-1 protein expression revealed a 40 kDa band, which is compatible with its cleavage by calpain (Figure 6D), an important marker of caspase-independent cell necrosis [35], while Caspase 3 gene expression was decreased in the same groups (data not shown). Assessment of p53 and p21 gene expression, two genes intimately related to cell senescence, showed that they both were augmented in the OLD group (Figure 6E,F), although HSD-fed rats presented increased expression only of the p21 gene, suggesting anticipated neuronal aging.

## 4. Discussion

The exponential rise of worldwide sucrose consumption during the last decades has been directly correlated to the epidemic growth of MetS and its associated dysfunctions, affecting individual quality of life and health insurance systems [36,37]. A positive correlation between sucrose-rich diet intake and its several deleterious metabolic outcomes, such as central obesity, dyslipidemia, nonalcoholic fatty liver disease, and IR, has been consistently shown [38,39,40,41,42]. Besides its peripheral metabolic damages, MetS has also been classified as an age-related illness with an important impact on cognitive decline in both humans and rodents [4,9].

We have recently shown that rats fed a high-sucrose diet upon weaning presented MetS along with motor and behavioral, but not cognitive, impairments at the young age of 3 months old, which were ascribed to hippocampal ER stress [23]. Thus, in this study, we expanded those findings by showing that rats’ exposure to the same diet until adulthood (6 months old) not only aggravated metabolic dysfunctions and serum oxidative profile, but worsened hippocampus-driven neuronal functions, especially cognition, as a consequence of the precocious failure of UPR pro-adaptive responses as well as increased expression of neuronal aging and cell death markers.

Long-term exposure of HSD rats to a high-sucrose diet until 6 months old deepened the obesogenic phenotype, characterized by increased body weight, stronger central obesity, hepatic steatosis, hypertriglyceridemia-based dyslipidemia, dysglycemia, hyperinsulinemia, and IR, in accordance with previous data from us and others [23,38,39,43]. These alterations might be primarily attributed to the longer consumption of a high-sucrose diet, since glucose elicits insulin release toward its own uptake by insulin-sensitive tissues, whereas fructose is promptly uptaken by hepatic GLUT-2 in an insulin-independent manner [44]. In the liver, both glucose and fructose are partially converted into fatty acids through de novo lipogenesis, favoring triglyceride synthesis and VLDL secretion [45]. Such an effect has been shown to be exacerbated upon co-ingestion, for instance as sucrose [46]. On the other hand, one must have in mind that the lower dietary protein content in our HSD may have also played a role, since it has been shown that diets containing lower protein levels also lead to increased body weight, adiposity, and other metabolic outcomes, such as hepatic steatosis [47,48,49].

Systemic oxidative stress is an essential clinical manifestation of MetS, leading to chronic metabolic dysfunction associated to cellular senescence and neurological disorders [50]. HSD animals presented serum oxidative stress characterized by increased SOD and CAT but decreased GPx activity, resulting in higher lipid peroxidation. Under chronic oxidative stress, GPx has been shown to lower its activity prior to SOD and CAT [51]. Moreover, obese rats present decreased GPx activity subsequent to reduced glutathione synthesis [52]. Unsolved oxidative stress impacts cell fate, causing widespread apoptosis and necrosis [53] in consequence to direct DNA damage and telomere shortening [54]. Likewise, long-term survival as well as hippocampal neuronal turnover are compromised, leading to cognitive impairment [53].

In addition to MetS development, our adult HSD rats showed motor and cognitive impairment compatible with that observed in OLD rats. Motor deficit in rotarod test is generally associated with increased body mass, skeletal muscle atrophy—both found in our adult HSD rats—as well as motor neuron degeneration [55]. Nevertheless, considering our previous report that young HSD rats displayed deficient performance on the rod in spite of having no increased body mass or muscle atrophy [23], we speculate that degeneration of motor-controlling brain areas would be the primary cause. This speculation is corroborated by findings that mice fed a high-fat diet for 10 months poorly performed on the rotarod in a way not correlated with adipose mass increase [56].

Concerning the assessment of cognitive functions, HSD rats had worse performance in a water maze test, in a fashion very similar to that of OLD rats, denoting impairment of hippocampus-dependent mechanisms responsible for spatial learning [57,58,59] and short-term memory retention [57,60,61,62,63]. Sucrose-induced obesity has been related to memory decline subsequent to hippocampal IR [57,58,64], which results from elevated glucose and triglyceride levels that penetrate the blood–brain barrier and interfere with insulin signaling [58]. Recently, sucrose intake’s deleterious effects on hippocampal-dependent memory were shown to be null in mice lacking insulin signaling in neuropeptide Y neurons [65]. Beyond hippocampal IR, other mechanisms such as reduced hippocampal dendritic spine density and impaired long-term synaptic potentiation [62], decreased neurogenesis [66], altered GABA-mediated neurotransmission [60], and locally elevated pro-inflammatory cytokines [61] may also play a role in cognitive decline associated to high sucrose intake.

ER stress pathways have emerged as a common mechanism underlying neuronal damage induced by oxidative stress, IR, and/or aging [19,20,67]. Our adult HSD rats presented remarkable hippocampal ER stress mainly characterized by failure of pro-adaptive signaling, as depicted by very low gene expression of UPR-sensors, chaperones, and Bdnf. On the other hand, gene expressions of Chop and p21, both markers of cell death and aging, were increased in HSD rats, whereas the anti-apoptotic factor Bcl2 was reduced and Nrf2 was unchanged. OLD rats presented a very similar profile, except for unchanged Chop and increased Nrf2 expressions.

The failure of UPR pro-adaptive pathways found in our adult HSD rats is in accordance with others who showed decreased gene/protein expression of pro-adaptive sensors Grp78, Grp94, and Xbp1 as well as a lower spliced/unspliced Xbp1 ratio in the hippocampi of 6-month-old diabetic *db*/*db* mice [68]. Similarly, hippocampi from 24-month-old non-obese Wistar rats displayed a failure of pro-adaptive signaling featured by decreased gene expression of Perk, Grp78, Pdi, and Calnexin, along with increased gene expression of Chop [69]. Studies conducted with middle-aged [70] and aged [71] rodents provide further evidence for the progressive failure of neuronal UPR pro-adaptive pathways as a hallmark of idiopathic neuronal aging. Although we have not assessed protein phosphorylation of the UPR-sensors, constituting a limitation of this study, our set of data is consistently supported by the abovementioned studies.

The scenario in our adult HSD rats demonstrates worse hippocampal damage, since they presented higher gene expression and protein levels of Chop as well as increased cleavage of Parp-1. Native whole Parp-1 is involved in the maintenance of genome integrity, but the event of the 40 kDa fragment demonstrated herein is consistent with its cleavage by calpain [35], a marker of necrosis and/or apoptosis in a caspase-independent way [72]. Concomitant increase of Chop expression and Parp-1 cleavage has also been found in neurodegenerative diseases such as Alzheimer’s disease [73]. All cognitive impairments found in both HSD and OLD groups seem to be intrinsically linked to the reduced expression of Bdnf, a capital factor for neurogenesis, cellular differentiation, and hippocampal plasticity that promotes Ire1α phosphorylation and Xbp1 splicing toward UPR-pro-adaptive responses [74,75,76]. Despite the absence of protein levels’ quantification, augmented gene expression of p21 allied to unchanged p53 in adult HSD rats supports the acceleration of hippocampal senescence and telomere shortening by sustained exposure to a high-sucrose diet and agrees with the demonstration of premature aging in obese *Agouti* mice [77].

The role of Nrf2 as a pivotal regulator of the intracellular antioxidant response and lifespan is consolidated [78]. A body of evidence has demonstrated a straight relationship between aging and Nrf2 downregulation [79,80], although opposite findings have displayed Nrf2 upregulation upon increased expression of p21, a well-known senescence marker [81]. In accordance with the latter, our OLD animals presented increased Nrf2 gene expression as compared to that of the other groups. It has also been demonstrated that increased Nrf2 protein expression restrains Chop overexpression in tunicamycin-induced ER stress [82], supporting the occurrence of elevated Nrf2 but unchanged Chop expression in OLD rats. On the other hand, hyperinsulinemia has been shown to independently promote Chop overexpression [83] as well as Nrf2 downregulation [84], corroborating the opposing pattern of Nrf2/Chop expression found in our hyperinsulinemic HSD in comparison with that in hypoinsulinemic OLD rats.

## 5. Conclusions

Collectively, the data herein presented originally show that early and sustained intake of a high-sucrose diet led to accelerated cognitive decline associated to MetS. Such cognitive impairment has been mainly characterized by increased expression of molecular markers of ER-stress-derived apoptosis, necrosis, and cellular senescence in the hippocampus subsequent to the failure of UPR pro-adaptive pathways, although the lack of a histological approach constitutes a limitation to be addressed in future studies. Furthermore, it corroborates the hypothesis that obesity and correlated metabolic disturbances hasten cellular aging by promoting ROS overgeneration, antioxidant defense insufficiency, and ultimately DNA damage and aging [12], to which we add UPR ineptitude as an underlying mechanism. Finally, this study has successfully established a direct relationship between cognitive decay and MetS-driven hippocampal ER stress, shedding light into novel mechanistic pathways and opening new venues for pharmacological or dietary interventions to treat and prevent premature aging caused by MetS.

## Data Availability

Data is contained within the article and Supplementary Material.

## Supplementary Materials

The following supporting information can be downloaded at: https://www.mdpi.com/article/10.3390/antiox11071395/s1, Figure S1: Full-length gels/blots for Grp94, Grp78 and Calreticulin; Figure S2: Full-length gels/blots for Pdi; Figure S3: Full-length gels/blots for Chop; Figure S4: Full-length gels/blots for Parp-1; Table S1: Primers sequences; Table S2: Morphometric and biochemical profile of OLD animals.
