# Supplementary material for: Hippocampal Endoplasmic Reticulum Stress Hastens Motor and Cognitive Decline in Adult Male Rats Sustainedly Exposed to High-Sucrose Diet
Source: Antioxidants (Basel). 2022 Jul 19;11(7):1395. doi: 10.3390/antiox11071395 (PMC9311607; doi:10.3390/antiox11071395)
Supplement: Supplementary file 1 [file antioxidants-11-01395-s001.zip › antioxidants-1797126-SI.pdf]

ORIGINAL RESEARCH

Hippocampal endoplasmic reticulum stress hastens motor and cognitive decline in adult male rats sustainedly exposed to high-sucrose diet.

AUTHORS

Bruno Araújo Serra Pinto[1], Thamys Marinho Melo[1], Karla Frida Torres Flister[1], Lucas Martins França[1], Vanessa Ribeiro Moreira[2], Daniela Kajihara[3], Nelmar de Oliveira Mendes[1], Silma Regina Pereira[2], Francisco Rafael Martins Laurindo[3], Antonio Marcus de Andrade Paes[1]*.

SUPPLEMENTARY INFORMATION

Supplementary Table S1. Primers sequences.

**Genes***	***Foward primer***	***Reverse primer***	***Amplicon***	**GenBank nº**
Gapdh	5'-GAGACAGCCGCATCTTCTTGT-3'	5'-CGACCTTCACCATCTTGTCTATGA-3'	62	NM_017008.4
Ire1α	5'-GGATGTGAGTGACCGAATAGAAAA-3'	5'-TCCAACTGCCGCACGAT-3'	60	NM_001191926.1
Perk	5'-GGCTGGTGAGGGATGGTAAA-3'	5'-TTGGCTGTGTAACTTGTGTCATCA-3'	64	NM_031599.2
Atf6	5'-TTCTCTGATGGCCGTGCAT-3'	5'-TGAAGATGACCCACAGAACCAA-3'	65	NM_001107196.1
Bdnf	5'-CACTTTTGAGCACGTGATCGA-3'	5'-AGAAGGTTCGGCCCAACG-3'	59	NM_001270630.1
Nrf2	5'-CCCATTGAGGGCTGTGATCT-3'	5'-GCCTTCAGTGTGCTTCTGGTT-3'	60	NM_031789.2
Grp94	5'-TGATGATGAAGCCGCAGTAG-3'	5'-AAGTTCCCAGTCCCACACAG-3'	88	NM_001012197.2
Grp78	5'-AAAGAAGGTCACCCATGCAGTT-3'	5'-GCCGCTGTGCATCATTGA-3'	59	NM_013083.2
Pdi A2	5'-GCACCTGGTCACAGAATTCAAC-3'	5'-TGAGGATCCTGGCTGCAAA-3'	62	NM_001105775.2
Calreticulin	5'-GCCAGACACTGGTGGTACAGTTC-3'	5'-AATATCGACTGTGGGGGCG-3'	60	NM_022399.2
Bcl2	5'-GGGATGCCTTTGTGGAACTATATG-3'	5'-CAGCCAGGAGAAATCAAACAGA-3'	62	NM_016993.1
Chop	5'-TGGCACAGCTTGCTGAAGAG-3'	5'-TCAGGCGCTCGATTTCCT-3'	54	NM_001109986.1
p53	5'-AAGACCAAGAAGGGCCAGTCTA-3'	5'-CAATGATCAAGAAAGTGGGGC-3'	61	NM_030989.3
p21	5'-CCTCCGCTGGGAACGTT-3'	5'-GCATAACTTCTGCTCAAGCACG-3'	56	NM_031550.1

* Gapdh: Glyceraldehyde-3-phosphate dehydrogenase; Ire1α: Inositol-requiring enzyme 1 alpha; Perk: Protein kinase RNA-like ER kinase; Atf6: Activating transcription factor 6; Bdnf: Brain derived neurotrophic factor; Nrf2: Nuclear factor, erythroid derived 2, like 2; Grp94: Glucose regulated protein 94; Grp78: Glucose regulated protein 78; Pdi A2: Protein disulfide isomerase family A, member 2; Calreticulin; Bcl2: B-cell CLL/lymphoma 2; Chop: C/EBP-homologous protein; p53: Tumor protein p53; p21: Cyclin dependent kinase inhibitor 1A.

Supplementary Table S2. Morphometric and biochemical profile of OLD animals.

Parameters	OLD group	CTR group	*p* value
**Body weight**	572.7 ± 15.78 g	422.4 ± 5.39 g	<0,0001
**Fasting glycemia**	83.4 ± 2.5 mg/dL	93.1 ± 1.1 mg/dL	0,0075
**Fed glycemia**	104.3 ± 1.72 mg/dL	102.6 ± 3.4 mg/dL	0,6673
**Insulinemia**	0.09 ± 0.02 µLU/mL	0.32 ± 0.04 µLU/mL	0,0009
**HOMA-B**	1.17 ± 0.36	3.12 ± 0.36	0,0050
**HOMA-IR**	0.0008 ± 0.0002	0.003 ± 0.0003	0,0007
**Retroperitoneal fat pad**	2.27 ± 0.2 g/100 BW	0.96 ± 0.1 g/100 BW	0,0004
**Periepididymal fat pad**	2.52 ± 0.22 g/100 BW	1.15 ± 0.09 g/100 BW	0,0003
**Mesenteric fat pad**	2.66 ± 0.21 g/100 BW	0.75 ± 0.08 g/100 BW	<0,0001

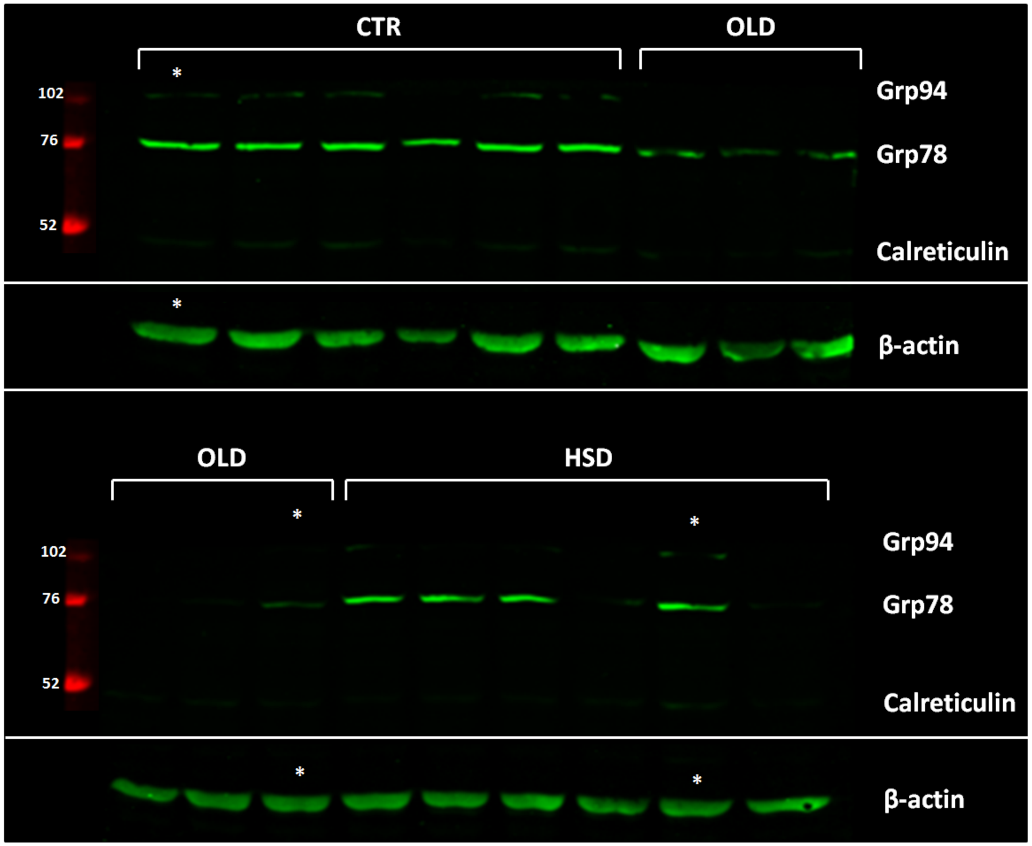

Supplementary Figure S1. Full-length gels/blots for Grp94, Grp78 and Calreticulin. Protein levels of Grp94, Grp78, Calreticulin and β-Actin assessed in hippocampus of rats fed a standard chow (CTR) and high-sucrose diet (HSD) until 6-months-old from weaning, and middle-aged (20-months-old) animals fed a standard chow (OLD). * indicate the bands used as representative in Figures 5J, K and M.

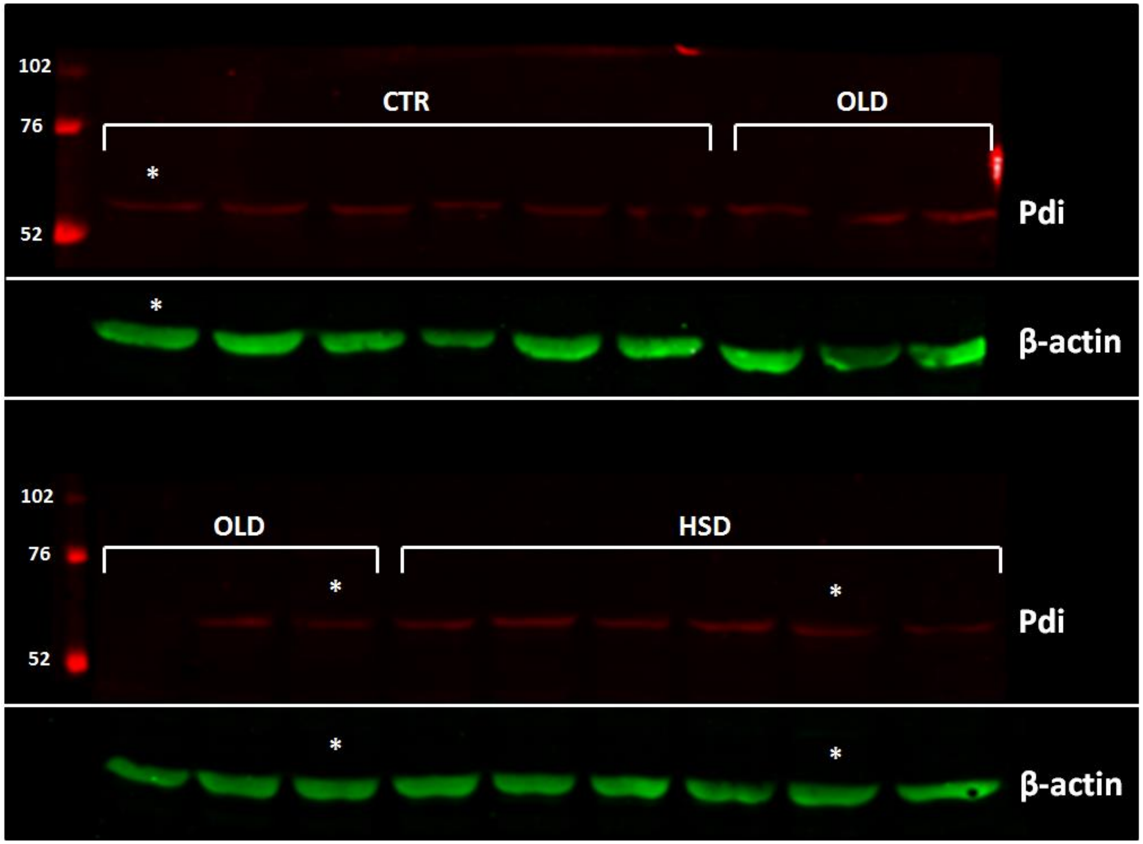

Supplementary Figure S2. Full-length gels/blots for Pdi. Protein levels of Pdi and β-Actin assessed in hippocampus of rats fed a standard chow (CTR) and high-sucrose diet (HSD) until 6-months-old from weaning, and middle-aged (20-months-old) animals fed a standard chow (OLD). * indicate the bands used as representative in Figure 5L.

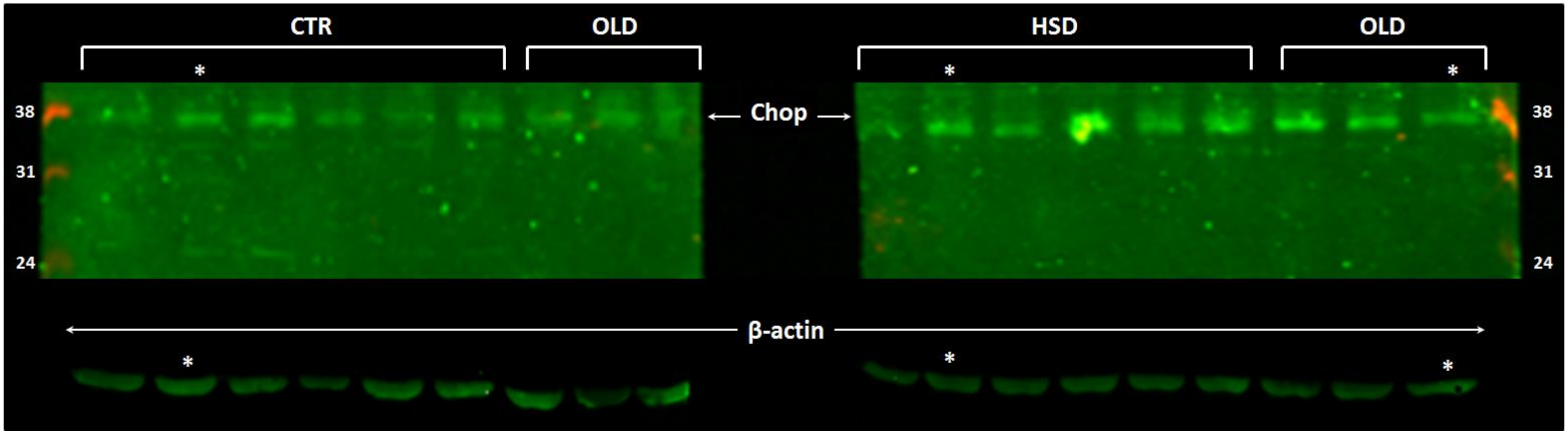

Supplementary Figure S3. Full-length gels/blots for Chop. Protein levels of Chop and β-Actin assessed in hippocampus of rats fed a standard chow (CTR) and high-sucrose diet (HSD) until 6-months-old from weaning, and middle-aged (20-months-old) animals fed a standard chow (OLD). * indicate the bands used as representative in Figure 6C.

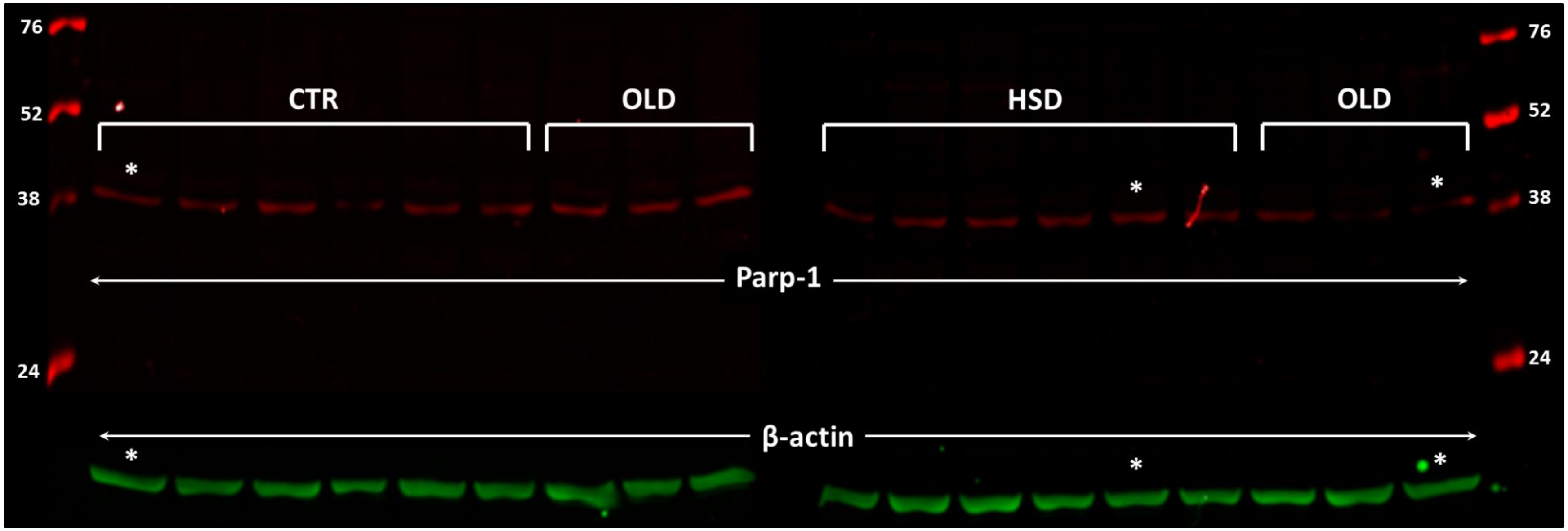

Supplementary Figure S4. Full-length gels/blots for Parp-1. Protein levels of Parp-1 and β-Actin assessed in hippocampus of rats fed a standard chow (CTR) and high-sucrose diet (HSD) until 6-months-old from weaning, and middle-aged (20-months-old) animals fed a standard chow (OLD). * indicate the bands used as representative in Figure 6D.
